# Safety of Tepotinib in Patients With *MET* Exon 14 Skipping NSCLC and Recommendations for Management

**DOI:** 10.1016/j.cllc.2022.03.002

**Published:** 2022-03-17

**Authors:** Remi Veillon, Hiroshi Sakai, Xiuning Le, Enriqueta Felip, Alexis B. Cortot, Egbert F. Smit, Keunchil Park, Frank Griesinger, Christian Britschgi, Yi-Long Wu, Barbara Melosky, Shobhit Baijal, Gilberto de Castro, Michaela Sedova, Karin Berghoff, Gordon Otto, Paul K. Paik

**Affiliations:** 1 CHU Bordeaux, Service des Maladies Respiratoires, Bordeaux, France; 2 Department of Thoracic Oncology, Saitama Cancer Center, Saitama, Japan; 3 Department of Thoracic Head and Neck Medical Oncology, The University of Texas MD Anderson Cancer Center, Houston, TX; 4 Department of Oncology, Vall d’Hebron Institute of Oncology (VHIO), Barcelona, Spain; 5 Univ. Lille, CHU Lille, CNRS, Inserm, Institut Pasteur de Lille, UMR9020 – UMR-S 1277 - Canther, F-59000 Lille, France; 6 Netherlands Cancer Institute, Amsterdam, The Netherlands; 7 Samsung Medical Center, Sungkyunkwan University School of Medicine, Seoul, Republic of Korea; 8 Pius-Hospital, University Medicine Oldenburg, Department of Hematology and Oncology, University Department Internal Medicine-Oncology, Oldenburg, Germany; 9 Department of Medical Oncology and Hematology, Comprehensive Cancer Center Zurich, University Hospital Zurich, Zurich, Switzerland; 10 Guangdong Lung Cancer Institute, Guangdong Provincial People’s Hospital and Guangdong Academy of Medical Sciences, Guangzhou, China; 11 Medical Oncology, The University of British Columbia, Vancouver, Canada; 12 University Hospitals Birmingham NHS Foundation Trust, Birmingham, UK; 13 Department of Clinical Oncology, Hospital Sírio-Libanês, São Paulo, Brazil; 14 Cytel Czech Republic, s.r.o., Prague, Czech Republic; 15 Global Patient Safety, the healthcare business of Merck KGaA, Darmstadt, Germany; 16 Global Clinical Development, the healthcare business of Merck KGaA, Darmstadt, Germany; 17 Thoracic Oncology Service, Memorial Sloan-Kettering Cancer Center, New York, NY; 18 Department of Medicine, Weill Cornell Medical College, New York, NY

**Keywords:** Adverse event, MET inhibitor, Non-small cell lung cancer, Edema, Nausea

## Abstract

To further characterize the safety of tepotinib in patients with *MET* exon 14 skipping non-small cell lung cancer, we analyzed adverse events of clinical interest in the phase II VISION trial (N = 255). The most frequent adverse events were largely mild/moderate and manageable with supportive measures and/or dose reduction/interruption, and caused few withdrawals.

**Introduction:**

The MET inhibitor tepotinib demonstrated durable clinical activity in patients with advanced *MET* exon 14 (*MET*ex14) skipping NSCLC. We report detailed analyses of adverse events of clinical interest (AECIs) in VISION, including edema, a class effect of MET inhibitors.

**Patients and Methods:**

Incidence, management, and time to first onset/resolution were analyzed for all-cause AECIs, according to composite categories (edema, hypoalbuminemia, creatinine increase, and ALT/AST increase) or individual preferred terms (pleural effusion, nausea, diarrhea, and vomiting), for patients with *MET*ex14 skipping NSCLC in the phase II VISION trial.

**Results:**

Of 255 patients analyzed (median age: 72 years), edema, the most common AECI, was reported in 69.8% (grade 3, 9.4%; grade 4, 0%). Median time to first edema onset was 7.9 weeks (range: 0.1–58.3). Edema was manageable with supportive measures, dose reduction (18.8%), and/or treatment interruption (23.1%), and rarely prompted discontinuation (4.3%). Other AECIs were also manageable and predominantly mild/moderate: hypoalbuminemia, 23.9% (grade 3, 5.5%); pleural effusion, 13.3% (grade ≥ 3, 5.1%); creatinine increase, 25.9% (grade 3, 0.4%); nausea, 26.7% (grade 3, 0.8%), diarrhea, 26.3% (grade 3, 0.4%), vomiting 12.9% (grade 3, 1.2%), and ALT/AST increase, 12.2% (grade ≥ 3, 3.1%). GI AEs typically occurred early and resolved in the first weeks.

**Conclusion:**

Tepotinib was well tolerated in the largest trial of a MET inhibitor in *MET*ex14 skipping NSCLC. The most frequent AEs were largely mild/moderate and manageable with supportive measures and/or dose reduction/interruption, and caused few withdrawals in this elderly population.

## Introduction

Approximately 3% to 4% of patients with non–small cell lung cancer (NSCLC) have tumors harboring *MET* exon 14 (*MET*ex14) skipping alterations.^1–4^ With a median age of approximately 72 years, these patients are typically older than those with other driver mutations, and more often have a history of smoking.^5^ *MET*ex14 skipping is a negative prognostic factor predicting poorer survival by multivariate analysis (HR, 2.156; 95% CI: 1.096, 4.242; *P* = .026) compared with patients with NSCLC without *MET*ex14 skipping alterations.^6^ However, these tumors are responsive to tyrosine kinase inhibitors (TKIs) that target the MET receptor.^2^

Tepotinib is an oral, potent, and highly specific MET TKI with a pharmacokinetic profile that enables once daily (QD) dosing.^7,8^ In the phase II VISION trial, tepotinib 500 mg (450 mg active moiety) QD demonstrated durable clinical activity in advanced *MET*ex14 skipping NSCLC, with an objective response rate of 49.1%, and a median duration of response of 13.8 months.^9–12^ Overall health-related quality of life (QoL) was maintained during tepotinib treatment.^9,13^ Based on this study, tepotinib has been approved for the treatment of advanced/metastatic NSCLC with *MET*ex14 skipping in several countries, and is recommended in clinical guidelines.^14–18^

Comprehensive safety evaluation of novel agents is essential to enable thorough characterization of the benefit–risk profile, including adverse events (AEs) with the potential to impact QoL. Such analyses can also provide valuable information to support physicians in managing AEs in clinical practice, especially as they gain experience with newer drug classes, such as selective MET TKIs. Furthermore, safety considerations are particularly relevant for patients with *MET*ex14 skipping NSCLC, who, due to their advanced age, may be more susceptible to AEs, frequently have comorbidities, and often require multiple concomitant medications.^6,19^

In phase I and II trials, tepotinib was administered at doses of up to 1400 mg/day without dose-limiting toxicity and was generally well tolerated at the 500 mg dose in patients with advanced solid tumors, NSCLC, or hepatocellular carcinoma.^8,9,20–22^ In VISION, tepotinib demonstrated a manageable safety profile in the largest dataset available so far from a clinical trial of a MET TKI in patients with *MET*ex14 skipping NSCLC.^9,11^ Treatment-related AEs were mostly mild or moderate and there was a relatively low rate of treatment discontinuation due to AEs.

To provide further information on the safety of tepotinib that is relevant for its use in clinical practice in patients with *MET*ex14 skipping NSCLC, we present detailed analyses of AEs of clinical interest in VISION, including incidence in subgroups, time to first onset, time to resolution, and management through treatment modification.

## Materials and Methods

### Study Design

VISION is a multicenter, multicohort, open-label, phase II trial evaluating tepotinib for the treatment of advanced NSCLC with *MET* alterations (clinicaltrials.gov identifier: NCT02864992).^9^ Patients with *MET*ex14 skipping were eligible for enrollment into Cohort A (pivotal cohort) and Cohort C (confirmatory cohort).

VISION was conducted in accordance with the Declaration of Helsinki, International Council on Harmonization Good Clinical Practice, local laws, and relevant regulatory requirements. The trial protocol was approved by an Independent Ethics Committee or Institutional Review Board at each site before study participation. Patients provided written informed consent.

### Patients

Eligible patients were aged ≥ 18 years and had an Eastern Cooperative Oncology Group performance status (ECOG PS) of 0 or 1, and histologically and/or cytologically confirmed, measurable, advanced NSCLC of any histology, with *MET*ex14 skipping alterations detected by liquid, and/or tissue biopsy.^9^ Up to 2 prior lines of therapy were allowed and could include immunotherapies, but not agents targeting the MET/hepatocyte growth factor (HGF) pathway.

### Treatment

Patients received tepotinib 500 mg QD orally with food until disease progression, consent withdrawal, or discontinuation due to AEs. To manage AEs, investigators could reduce the tepotinib dose or temporarily interrupt tepotinib until the AE recovered to grade ≤ 2 or baseline values. The reduced dose was initially 300 mg (270 mg active moiety), which could be reduced further to 200 mg (180 mg active moiety) if required. In Protocol v8 (release date: January 17, 2020), this was simplified to a single dose reduction to 250 mg (225 mg active moiety). According to translational pharmacokinetic/pharmacodynamic modelling,^23^ the 250 mg dose is predicted to maintain biologically meaningful (≥ 95%) MET inhibition in > 80% of the population.

After dose reduction, the dose could be re-escalated to 500 mg at the investigator’s discretion, but there was no limit to the duration of reduced-dose treatment. The maximum permitted duration of continuous treatment interruption was 21 days, but the number of interruptions, and/or cumulative time off treatment were not limited. After treatment interruption, tepotinib was resumed at 500 mg (or at the reduced dose, on a case-by-case basis according to investigator decision). Permanent treatment discontinuation was considered for AEs that did not resolve after dose reduction or treatment interruption. Patients who discontinued due to AEs continued to undergo tumor assessments until disease progression or consent withdrawal.

### Safety Evaluation

Safety assessments were performed at baseline, during treatment, at end of treatment, and at a safety follow-up visit 30 days after the end of treatment. Serious adverse events (SAEs) ongoing thereafter were followed up until stabilization or known outcome. AEs were graded according to the National Cancer Institute Common Terminology Criteria for Adverse Events v4.03, and coded according to the Medical Dictionary for Regulatory Activities (MedDRA) v23.0. Treatment-emergent AEs were defined as AEs that were absent before treatment, or worsened relative to before treatment, with onset occurring between the first dose, and 30 days after the last dose. Investigators assessed AEs as being either unrelated or related to tepotinib, based on factors including temporal relationship between the AE and drug administration, known adverse drug reactions, medical history, concomitant medication, course of the underlying disease, and trial procedures. Where pleural effusions were punctured, cytology results may have informed investigator assessment of pleural effusion causality, but did not impact on whether the event was recorded as an AE.

### Statistics

Treatment-emergent AEs were analyzed in all patients with *MET*ex14 skipping (ie in Cohorts A and C) who received at least 1 dose of tepotinib at the data cut-off (July 1, 2020). AE incidence was summarized using descriptive statistics. AEs of clinical interest included nausea, diarrhea, vomiting, and pleural effusion, and the composite events of edema, hypoalbuminemia, creatinine increase, and alanine aminotransferase (ALT) and/or aspartate aminotransferase (AST) increase (Supplementary Table 1).

Time to first onset and time to resolution were analyzed for AEs of clinical interest irrespective of the causal relation to study treatment. Time to first onset was described by the median and range for observed AEs, not accounting for competing events. Time to resolution was analyzed using Kaplan–Meier methods in a descriptive manner. As a participant could experience more than 1 AE, each event has been taken independently for the analysis of time to resolution.

## Results

### Patient Population

At the data cut-off (July 1, 2020), 255 patients with *MET*ex14 skipping had received tepotinib and were included in the analysis. Patients were generally elderly, with a median age of 72 years (range: 41–94). A total of 132 patients (51.8%) were female, 171 (67.1%) were white, and 72 (28.2%) were Asian. Seventy-one patients (27.8%) had ECOG PS 0, 184 (72.2%) had ECOGPS 1, and 121 (47.5%) had a history of smoking (Table 1).

The median duration of tepotinib treatment was 5.1 months (range: < 0.1–43.2) and treatment was ongoing in 101 patients (39.6%).

### Overall Safety Profile

**Figure 1:**
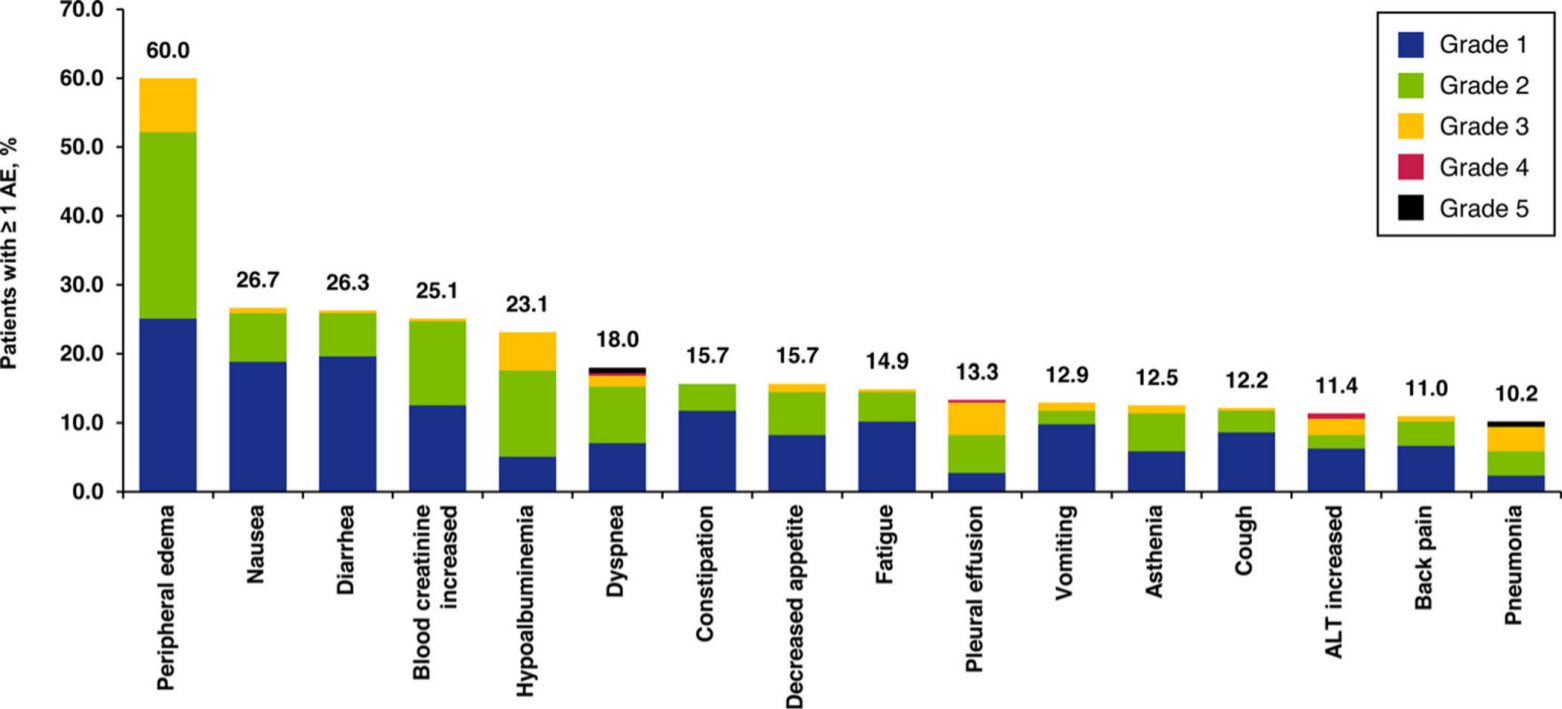
Incidence of all-cause AEs occurring at any grade in ≥ 10% of patients (preferred terms). Abbreviations: AE = adverse event; ALT = alanine aminotransferase

All-cause AEs were reported for 246 patients (96.5%) at any grade and 135 patients (52.9%) at grade ≥ 3 (Figure 1). The most common preferred terms were peripheral edema (60.0%; grade 3, 7.8%), nausea (26.7%; grade 3, 0.8%), diarrhea (26.3%; grade 3, 0.4%), blood creatinine increase (25.1%; grade 3, 0.4%), and hypoalbuminemia (23.1%; grade 3, 5.5%). The majority of the most frequent AEs were considered to be treatment-related. Preferred terms within the system organ class ‘eye disorders’ were reported in 14.9% of patients (grade 3, 0.4%). All-cause SAEs were reported for 115 patients (45.1%), and most commonly included were pleural effusion (17 patients, 6.7%), pneumonia (12 patients, 4.7%), and disease progression (12 patients, 4.7%).

A total of 140 patients (54.9%) had all-cause AEs leading to treatment modification, including dose reduction (76 patients; 29.8%), treatment interruption (112 patients; 43.9%), and permanent discontinuation (52 patients; 20.4%). As shown in Supplementary Figure 1, 123 patients (48.2%) had dose reduction and/or treatment interruption. Thirty-five patients (13.7%) with AEs leading to dose reduction and/or treatment interruption also had AEs leading to discontinuation. Recommendations and considerations for dose reductions, interruptions or discontinuations are shown in Supplementary Table 2. Other than AEs of clinical interest (detailed below), the most common AEs leading to discontinuation were dyspnea (4 patients, 1.6%) and general physical health deterioration (4 patients, 1.6%) (Supplementary Table 3). Despite the fact that, according to the protocol, patients with disease progression had to be taken off treatment, there were some reports of disease progression being an AE leading to discontinuation (4 patients, 1.6%). Of 21 patients with ≥ 1 tumor assessment after discontinuation due to an AE, investigator-assessed response at the time of discontinuation was partial response in 7, stable disease in 10, and disease progression in 4 patients. Disease control (ie stable disease or better) was observed after discontinuation of tepotinib for 7 of 10 patients at ≥ 6 weeks, 4 of 5 at ≥ 12 weeks, and 3 of 3 at ≥ 18 weeks post-discontinuation.

Thirty patients (11.8%) had fatal AEs, which were considered to be treatment-related in 3 patients (1.2%). As reported elsewhere,^11^ the 3 fatal treatment-related events were: acute respiratory failure secondary to interstitial lung disease (ILD); severe worsening of dyspnea; and acute hepatic failure (which led to death after the patient had withdrawn consent for study participation; however, in the later follow-up information, the investigator downgraded this SAE from grade 5 [death related to AE] to grade 4 [life-threatening]).

### Edema

**Figure 2:**
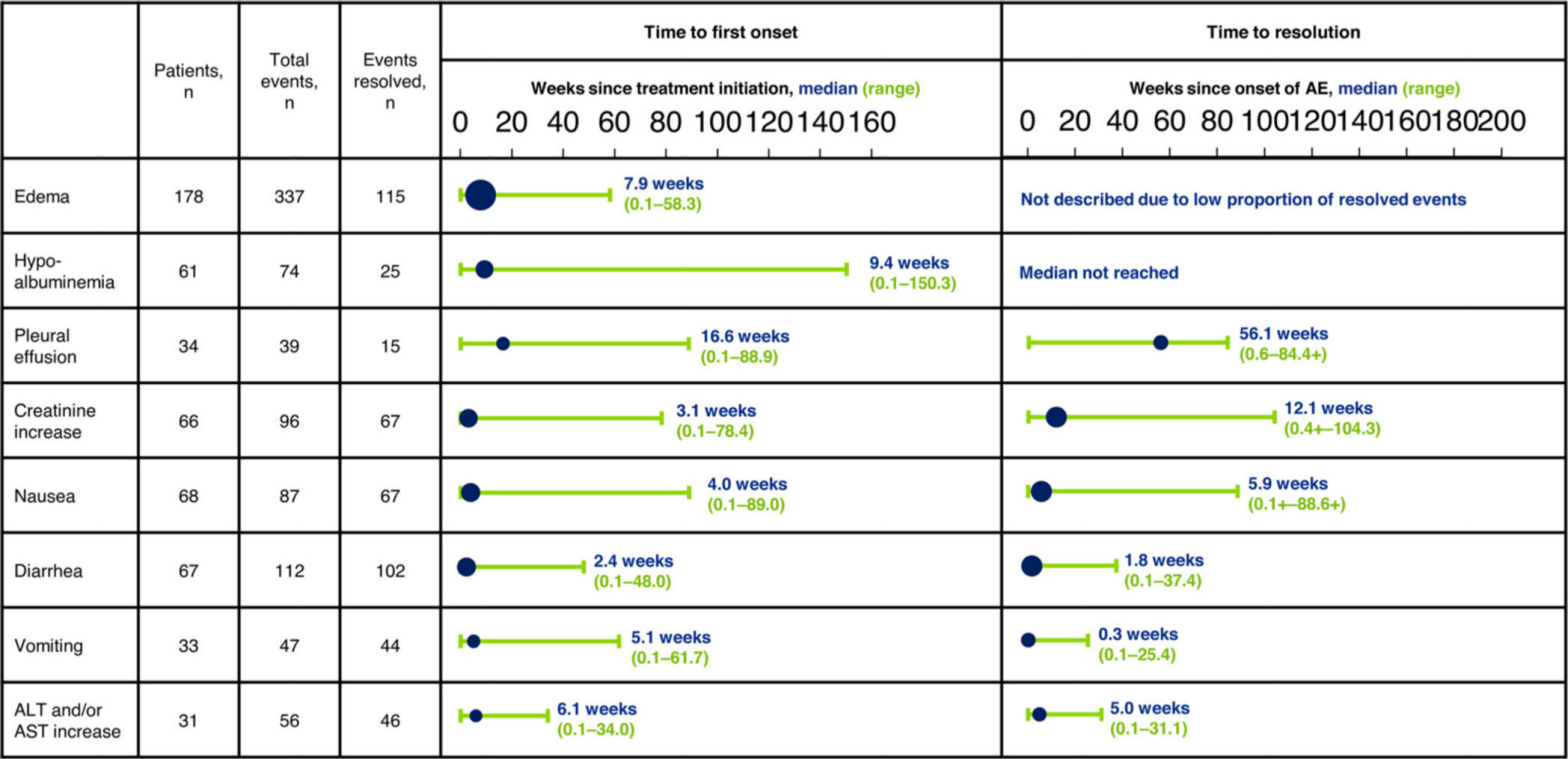
Time to first onset and time to resolution of AEs of clinical interest (all-cause). Plots indicate the median value (blue circles, size proportional to the number of patients) and range (green bars). The ‘+’ signs denote censored values. Abbreviations: AE = adverse event; ALT = alanine aminotransferase; AST = aspartate aminotransferase.

All-cause edema events (composite term) were reported for 178 patients (69.8%) and were mostly mild or moderate, with 70 patients (27.5%) and 84 patients (32.9%) having grade 1 and 2 events, respectively; 24 patients (9.4%) had grade 3 events and no grade ≥ 4 events occurred. By far the most common edema event was peripheral edema (153 patients; 60.0%) (Supplementary Table 4). Median time to first onset was 7.9 weeks (range: 0.1–58.3) for edema of any grade (Figure 2) and 18.9 weeks (range: 4.7–84.6) for grade 3 edema. At the time of the analysis, all edema events had resolved in 29 of 178 patients (16.3%). Due to the low proportion of patients with resolution of all edema events, median time to resolution is not regarded as meaningful and has not been described. Patients who develop edema stay longer on treatment. Compared to the overall population with a median duration of treatment of 22.3 weeks (range 0–188 weeks), patients with edema have a longer median duration of treatment of 29.6 weeks (range 0–188 weeks). However, this can be explained as the patients who stay longer on treatment are more likely to develop edema at some stage.

**Figure 3:**
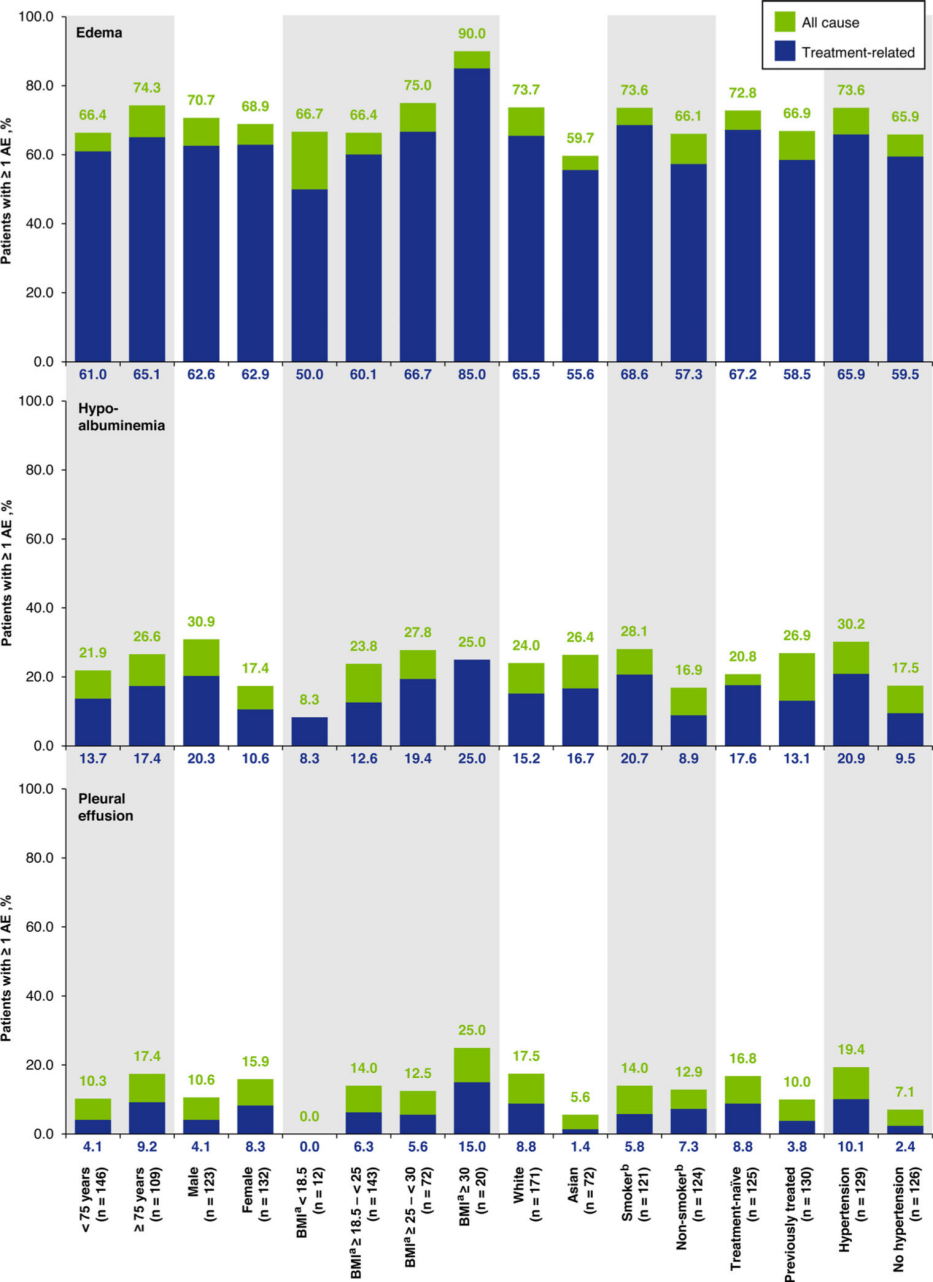
Incidence of edema (composite event), hypoalbuminemia (composite event) and pleural effusion, according to causality and patient subgroup. ^a^BMI was missing for 8 patients. ^b^Smoking history was missing for 10 patients. AE = adverse event; BMI = body mass index

Although generally consistent irrespective of patient characteristics, edema was more common in older patients, occurring in 148/202 patients (73.3%) aged ≥ 65 years versus 30/53 patients (56.6%) aged < 65 years, and in 81/109 patients (74.3%) aged ≥ 75 years versus 97/146 patients (66.4%) aged < 75 years. Edema rates were also higher in white versus Asian patients, and in patients with higher body mass index (BMI) (Figure 3). Edema led to dose reduction in 48 patients (18.8%), treatment interruption in 59 patients (23.1%), dose reduction and/or treatment interruption in 66 patients (25.9%), and permanent discontinuation in 11 patients (4.3%).

### Hypoalbuminemia

All-cause hypoalbuminemia (composite term) occurred in 61 patients (23.9%), of whom 14 patients (5.5%) had grade 1 events, 33 patients (12.9%) had grade 2 events, and 14 patients (5.5%) had grade 3 events (Supplementary Table 4). No grade ≥ 4 events were reported. Median time to first onset of hypoalbuminemia was 9.4 weeks (range: 0.1–150.3) (Figure 2). Of 74 hypoalbuminemia events, 25 (33.8%) had resolved at the time of the analysis, and median time to resolution had not been reached. Hypoalbuminemia incidence was mostly consistent across patient subgroups, but appeared to be more common in men versus women, smokers versus non–smokers, and patients with versus those without hypertension (Figure 3). Hypoalbuminemia led to dose reduction in 2 patients (0.8%), treatment interruption in 3 patients (1.2%), and dose reduction and/or treatment interruption in 4 patients (1.6%). No patients had hypoalbuminemia events leading to permanent discontinuation.

### Pleural Effusion

A total of 34 patients (13.3%) had all-cause pleural effusion. The highest grade of pleural effusion was grade 1 in 7 patients (2.7%), grade 2 in 14 patients (5.5%), grade 3 in 12 patients (4.7%), and grade 4 in 1 patient (0.4%). No grade 5 pleural effusion occurred. Median time to first onset of pleural effusion was 16.6 weeks (range 0.1–88.9) and median time to resolution was 56.1 weeks (range 0.6–84.4+) (Figure 2). The rate of pleural effusion appeared to be greater in white versus Asian patients and in patients with versus those without hypertension (Figure 3). Pleural effusion led to dose reduction in 7 patients (2.7%), treatment interruption in 11 patients (4.3%), dose reduction and/or treatment interruption in 14 patients (5.5%), and permanent discontinuation in 5 patients (2.0%).

### Creatinine Increase

**Figure 4:**
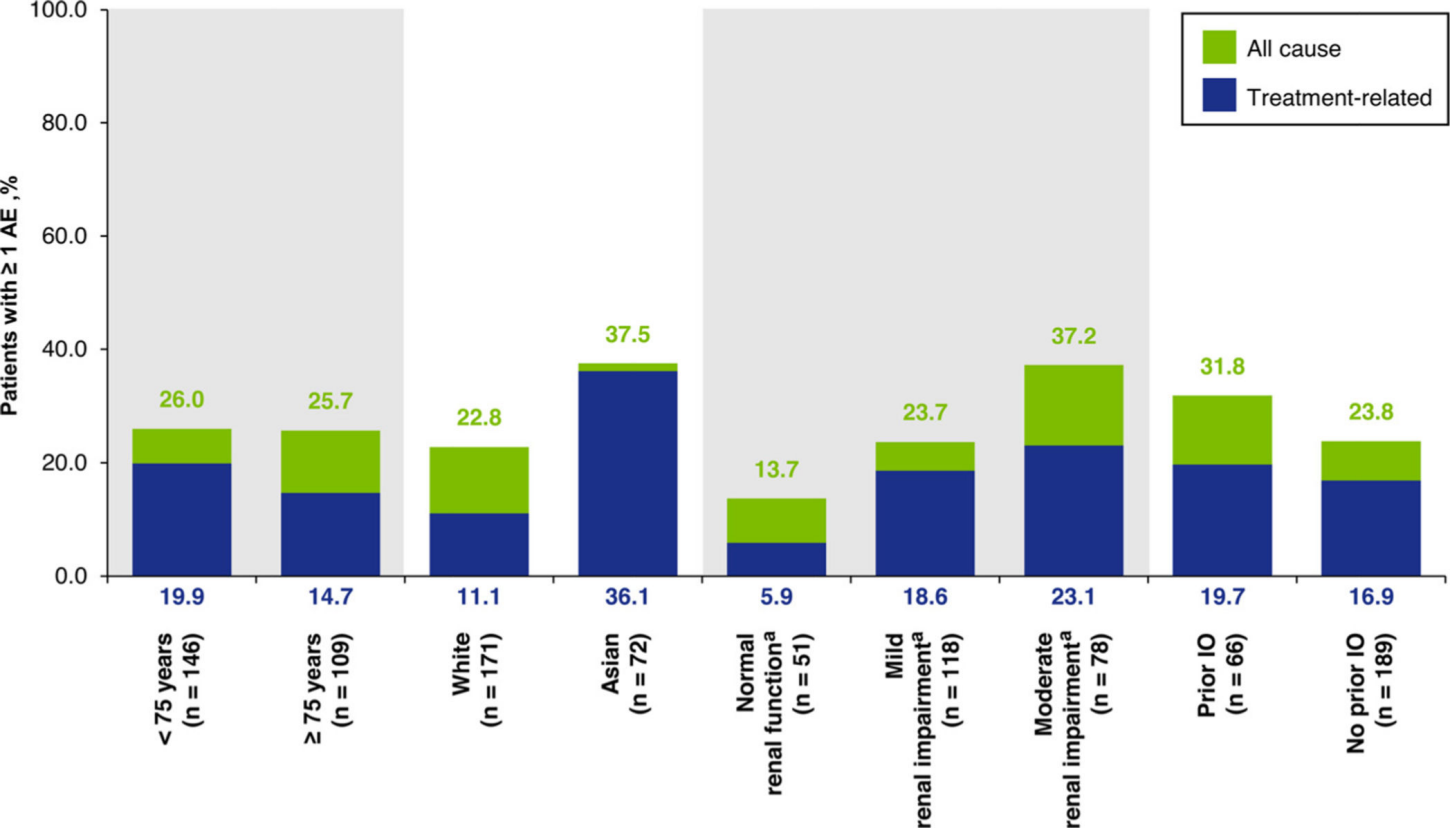
Incidence of creatinine increase (composite event), according to causality and patient subgroup. ^a^Renal impairment status was missing for 8 patients. Abbreviations: AE = adverse event; IO = immunotherapy buminemia, and 14 of 66 patients (21.2%) with creatinine increase did not have edema.

All-cause creatinine increase events (composite term) were reported in 66 patients (25.9%). These events were mild or moderate in all patients with 34 patients (13.3%) and 31 patients (12.2%) having grade 1 and 2 events, respectively; the only exception was 1 (0.4%) with a grade 3 event. Creatinine increases events comprised blood creatinine increased in 64 patients (25.1%; grade 3, 1 patient [0.4%]), and hypercreatininemia in 2 patients (0.8%) (Supplementary Table 4). Median time to first onset of creatinine increase was 3.1 weeks (range 0.1–78.4) and median time to resolution was 12.1 weeks (range 0.4+–104.3) (Figure 2). Incidence of creatinine increase was generally similar across patient subgroups, but appeared to be more common in Asian than white patients (Figure 4). There was no evidence that creatinine increase was associated with renal impairment; however, creatinine increase was more common in patients who had mild or moderate renal impairment at baseline. Creatinine increase was managed with dose reduction in 7 patients (2.7%), treatment interruption in 16 patients (6.3%), dose reduction and/or treatment interruption in 16 patients (6.3%), and permanent discontinuation in 2 patients (0.8%).

### Overlap Between Non–gastrointestinal (GI) AEs

There was no clear association between edema, hypoalbuminemia, pleural effusion, and creatinine increase when analyzed irrespective of event timing (Supplementary Figure 2). Of 178 patients with edema, 125 (70.2%) did not have hypoalbuminemia and 88 (49.4%) did not have hypoalbuminemia, pleural effusion, or creatinine increase events at any time during follow-up. Similarly, 20 of 34 patients (58.8%) with pleural effusion did not have hypoal-buminemia, and 14 of 66 patients (21.2%) with creatinine increase did not have edema.

### Gastrointestinal AEs

**Figure 5:**
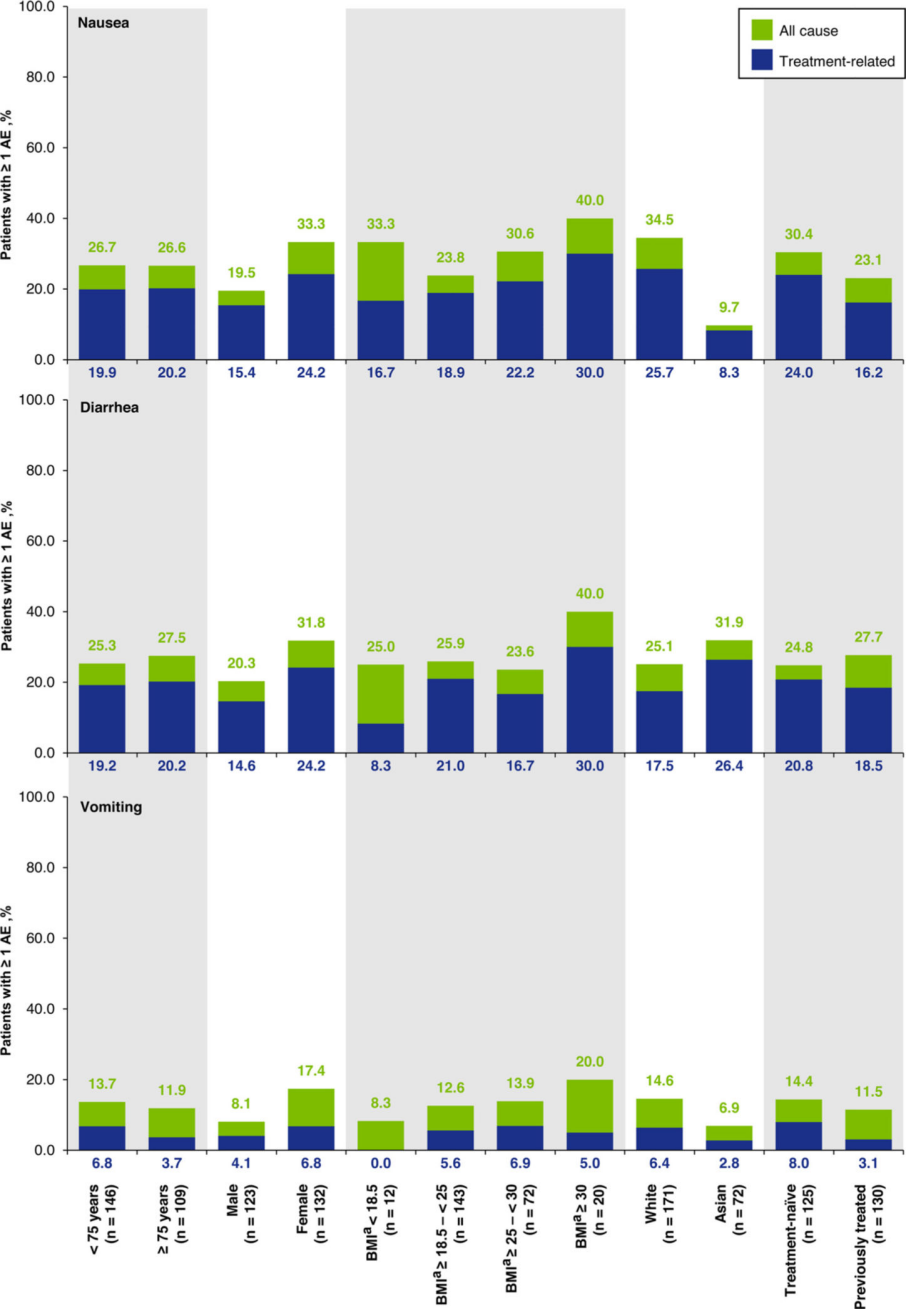
Incidence of nausea, diarrhea and vomiting, according to causality and patient subgroup. ^a^BMI was missing for 8 patients. Abbreviations: AE = adverse event; BMI = body mass index

All-cause nausea was reported in 68 patients (26.7%) and was mild or moderate in the vast majority of cases. Nausea was grade 3 in 2 patients (0.8%) and did not reach grade ≥ 4. Median time to first onset was 4.0 weeks (range 0.1–89.0) and median time to resolution was 5.9 weeks (range 0.1+–88.6+) (Figure 2). Nausea appeared to be more common in women, white patients, and patients with obesity (Figure 5). Nausea led to dose reduction in 2 patients (0.8%), treatment interruption in 5 patients (2.0%), dose reduction and/or treatment interruption in 6 patients (2.4%), and permanent discontinuation in 1 patient (0.4%).

All-cause diarrhea was reported in 67 patients (26.3%) and was mostly grade 1 (50 patients, 19.6%). Sixteen patients (6.3%) had grade 2 and 1 patient (0.4%) had grade 3 diarrhea. No grade ≥ 4 diarrhea occurred. Median time to first onset was 2.4 weeks (range 0.1–48.0) and median time to time to resolution was 1.8 weeks (range 0.1–37.4) (Figure 2). Diarrhea appeared to be more common in women versus men and in obese versus non–obese patients (Figure 5). Diarrhea led to dose reduction in no patients, treatment interruption in 5 patients (2.0%), and permanent discontinuation in 1 patient (0.4%).

All-cause vomiting was reported at any grade in 33 patients (12.9%) and was mild or moderate in all but 3 patients (1.2%), who had grade 3 vomiting. Median time to first onset was 5.1 weeks (range 0.1–61.7) and median time to resolution was 0.3 weeks (range 0.1–25.4) (Figure 2). The incidence of vomiting was generally consistent across patient subgroups, but appeared to be greater in women than men (17.4% vs. 8.1%) and in white patients versus Asian patients (14.6% vs. 6.9%) (Figure 5). Vomiting led to treatment interruption in 1 patient (0.4%) but did not prompt dose reduction or permanent discontinuation.

### ALT and/or AST Increase

**Figure 6:**
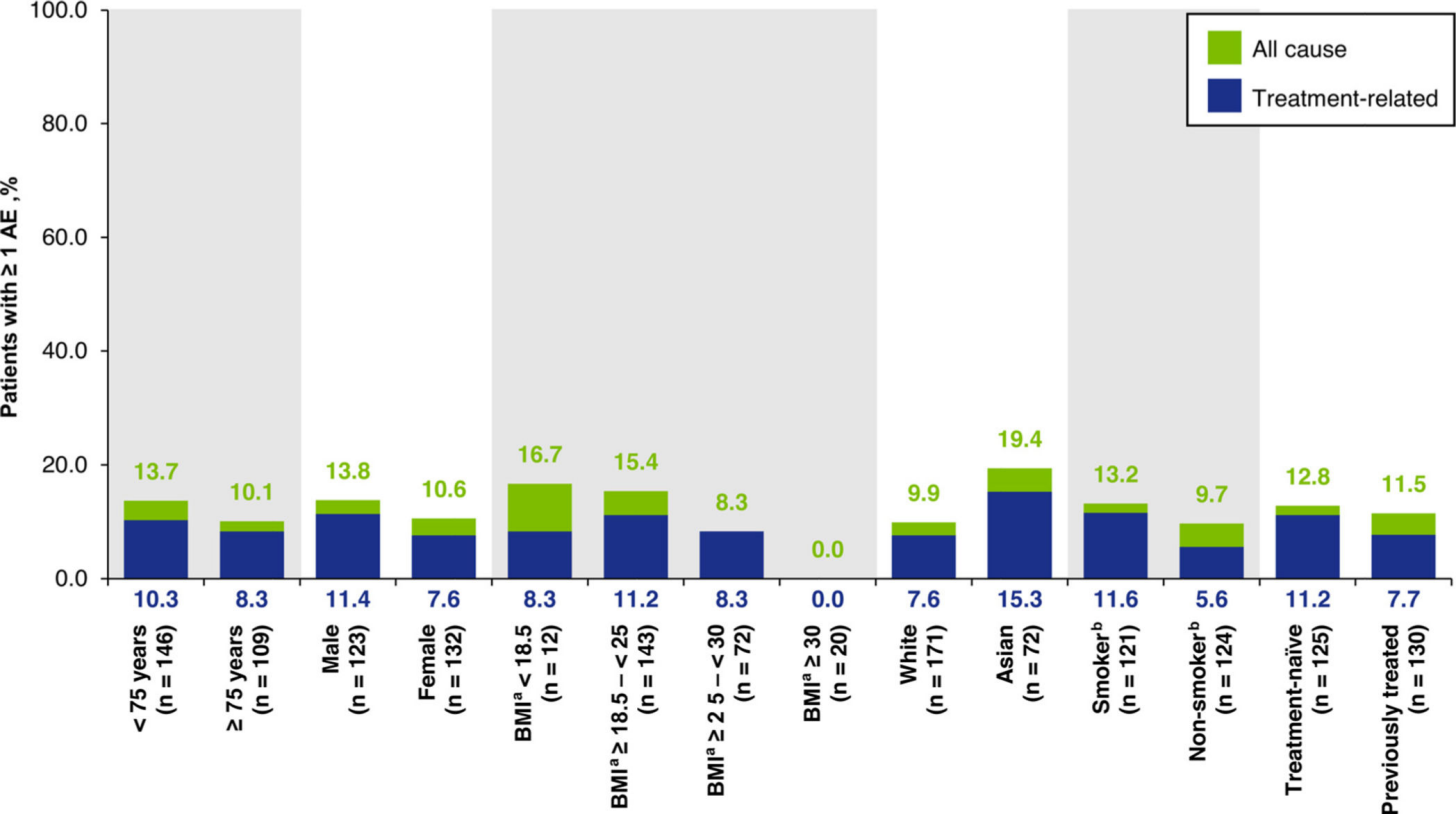
Incidence of ALT and/or AST increase (composite event), according to causality and patient subgroup. ^a^BMI was missing for 8 patients. ^b^Smoking history was missing for 10 patients. Abbreviations: AE = adverse event; ALT = alanine aminotransferase; AST = aspartate aminotransferase; BMI = body mass index

All-cause ALT and/or AST increase events (composite term) were reported for 31 patients (12.2%). Of these, 6 patients (2.4%) had grade 3 events and 2 (0.8%) had grade 4 events. The most common event was ALT increase (29 patients; 11.4%) (Supplementary Table 4). Median time to first onset of ALT and/or AST increase was 6.1 weeks (range 0.1–34.0), and median time to resolution was 5.0 weeks (range: 0.1–31.1) (Figure 2). ALT and/or AST increases were generally asymptomatic and occurred at a consistent rate across subgroups, although a potentially higher incidence was observed in Asian versus white patients (Figure 6). ALT and/or AST increase led to dose reduction in 2 patients (0.8%), treatment interruption in 9 patients (3.5%), and dose reduction and/or treatment interruption in 9 patients (3.5%). No patients had events leading to permanent discontinuation.

## Discussion

This analysis of the largest prospective trial to date of a MET inhibitor in patients with *MET*ex14 skipping NSCLC provides detailed information on the AE profile of tepotinib that can support its use in clinical practice. In this elderly population (which is representative of the median age of the NSCLC population harboring *MET*ex14 skipping alterations),^5^ tepotinib was well tolerated and demonstrated manageable safety, with a low frequency of treatment discontinuation due to AEs. Consistent with previous findings,^8,9,11,20–22^ the most common AEs included peripheral edema, nausea, diarrhea, blood creatinine increase and hypoalbuminemia, and were mostly mild or moderate. The good tolerability of tepotinib is also supported by patient-reported outcomes data indicating stability of global health status during the trial.^13^

The most frequent tepotinib AE was peripheral edema, which is observed with other agents targeting MET/HGF and is considered to be a class effect.^24,25^ In patients with *MET*ex14 skipping NSCLC, edema has been reported with capmatinib (peripheral edema: 59.8%, all-cause; 51.5%, treatment-related) or crizotinib (edema composite event: 50.7%, treatment-related).^24,25^ Although the underlying mechanism is unclear, peripheral edema may be explained by the role of MET signaling in protecting against vascular endothelial growth factor (VEGF)-induced endothelial hyperpermeability,^26^ for example, via downregulation of VEGF receptor-2.^27^ Another contributing factor in some patients may be hypoalbuminemia, which is also a potential MET inhibitor class effect.^28^ The pathophysiology of MET inhibitor-induced hypoalbuminemia is not well understood, but there was no evidence that hypoalbuminemia with tepotinib was secondary to hepatic dysfunction or renal loss. Edema incidence was largely consistent irrespective of patient characteristics and, although slow to resolve, was generally manageable. The considerably longer time to onset of grade 3 versus all-grade edema suggests that grade 3 edema develops slowly from lower-grade events. Although edema rates appeared to be higher in certain subgroups (eg older patients, white patients, and those with higher BMI), only advanced age was correlated with edema risk independent of tepotinib exposure in a recent safety–exposure analysis in patients with solid tumors.^29^

Although not life-threatening, edema can negatively impact QoL and may be much more difficult to manage once it becomes established. Prevention, early recognition, and prompt intervention are therefore critical for successful mitigation. As edema is not always immediately obvious or symptomatic, body weight and limb circumference should be proactively monitored at baseline and regularly during treatment to facilitate early recognition and intervention. Physical activity can be recommended for edema prevention. If peripheral edema occurs, it is important to exclude other causes, especially heart failure, given the advanced age of the population. Peripheral edema management options include support stockings, limb elevation, increased physical activity, and kinesiotherapy. Diuretics, such as furosemide, should be used with caution, as they have the potential to impair renal function^30^ and clinical experience suggests that they may provide only short-term relief. As peripheral edema is a class effect, switching to a different MET inhibitor is unlikely to be helpful. Dose reduction and/or treatment interruption, including frequent, short treatment breaks, appear to be the best current options for managing edema, and should be implemented early to limit severity. Cross-functional management in a lymphedema clinic can be considered.^31^

In VISION, 25.9% of patients had all-cause creatinine increase events, but there was no evidence for an association with renal impairment. Creatinine increases occurred early, were mild or moderate in all but 1 patient, and infrequently prompted treatment modification. Creatinine elevations were also common with capmatinib in *MET*ex14 skipping NSCLC in GEOMETRY mono-1 (34.0%).^25^ Rather than indicating renal toxicity, creatinine elevation is likely explained by direct inhibitory effects of tepotinib and capmatinib on renal tubular transporters,^32,33^ which has also been shown for other TKIs.^34^ Lack of close correlation with edema suggests creatinine increases are not a prerenal effect of edema. Frequent monitoring of creatinine levels is recommended during the first 2 months of treatment. As illustrated in a recent case report of a patient with increased creatinine during capmatinib treatment,^35^ alternative markers of glomerular filtration rate, such as blood urea nitrogen or cystatin C,^36^ can be considered to evaluate renal function independent of creatinine levels, and so avoid unnecessary dose modifications.

All-cause pleural effusion was reported in 13.3% of patients and did not appear to be secondary to hypoalbuminemia in a majority of patients. Importantly, pleural effusion may represent both treatment-related AEs, and complications of the underlying disease, including malignant pleural effusion.^37^ Thoracentesis with cytological assessment can help to differentiate malignant pleural effusion from paramalignant phenomena, caused by indirect effects of the tumor or its treatment.^37^ This practice was reflected in VISION by submission of thoracentesis cytology results from patients with pleural effusion to the independent review committee for consideration in response assessment. If malignant pleural effusion is excluded, other potential causes, such as prior chemotherapy or radiotherapy,^37^ should be considered before attributing the event to tepotinib.

The most common tepotinib AEs included nausea, diarrhea, and vomiting, which were predominantly mild or moderate, tended to resolve in the first weeks, and resulted in few treatment discontinuations. Nausea, diarrhea and vomiting are also frequent with other oral TKIs,^38–40^ including capmatinib^33^ (44%, 18%, and 28%, respectively, for all-cause AEs), crizotinib^24^ (41%, 39%, and 29%, for treatment-related AEs), and savolitinib^41^ (53%, not reported, and 33%, for all-cause AEs). As for other agents,^42^ GI tolerability may be improved by taking tepotinib with food, in line with the dosing recommendations.^14^ For GI symptom relief, standard antidiarrheals (eg loperamide) and antiemetics (eg 5-HT3 antagonists) are recommended, and temporary tepotinib interruption can be considered.

As with other oral TKIs,^38–40^ ALT and/or AST increase events were frequent with tepotinib. ALT/AST elevations were mostly mild or moderate, reversible, asymptomatic, and did not typically require treatment modification. Liver function tests are recommended every 2 weeks during the first 3 months of treatment and every month thereafter,^14^ but transaminase elevations do not generally require dose modification unless accompanied by symptoms.

As reported elsewhere,^11^ the incidence of ILD-like events in VISION was low. Time to onset and resolution was not analyzed due to the small number of events. ILD is a frequent comorbidity in lung cancer and can also be induced by anticancer therapies, including epidermal growth factor receptor inhibitors.^43,44^ As a significant and potentially fatal adverse reaction, ILD requires careful monitoring, especially in patients with risk factors, such as pre-existing ILD/pneumonitis, or recent prior immunotherapy or thoracic radiotherapy.^45^

The tepotinib safety profile contrasts with that of standard treatments frequently used in broader NSCLC patient populations. Unlike tepotinib, chemotherapy use is limited by frequent grade ≥ 3 hematologic toxicity with platinum-based doublets, and cumulative neurotoxicity and ototoxicity with cisplatin-containing regimens.^46,47^ Meanwhile, cancer immunotherapies have a distinct set of immune-related AEs, which affect a variety of organs, can be severe, and may have a delayed onset even after immunotherapy discontinuation.^48,49^ In a meta-analysis, immune-related AEs were reported in 22% of patients with NSCLC, and most commonly affected the endocrine, integumentary, pulmonary, and GI systems.^50^ However, for patients previously treated with these therapies, it is reassuring that tepotinib has a consistent treatment-related AE profile irrespective of therapy line or prior treatment type (including immunotherapies).^11^

Peripheral edema and nausea are the most frequent AEs associated with tepotinib, which is similar to the most common AEs of other oral MET TKIs used for the treatment of *MET*ex14 skipping NSCLC, such as capmatinib, savolitinib, or crizotinib.^51^ All 4 of these MET TKIs (tepotinib, capmatinib, savolitinib, or crizotinib) also have warnings for hepatotoxicity, with additional warnings of embryo-fetal toxicity with tepotinib; photo sensitivity and embryo-fetal toxicity with capmatinib; severe allergic reactions with savolitinib; and vision loss, QT interval prolongation, bradycardia, and embryo-fetal toxicity with crizotinib.^51^ While the incidence of eye disorders was low with tepotinib, vision disorders have been reported in 45% of patients treated with crizotinib.^24^

The present analysis provides insights into tepotinib safety according to patient characteristics, but subgroup data must be interpreted in light of the small size of some subgroups. Furthermore, time to first onset and time to resolution analyses were conducted for descriptive purposes only, including all AEs for all patients independently.

## Conclusion

In conclusion, in VISION, the largest and most comprehensive safety analysis of a MET inhibitor in patients with *MET*ex14 skipping NSCLC, tepotinib was well tolerated, with mostly mild to moderate AEs. The manageable safety profile observed, with few withdrawals due to AEs, is especially relevant for this elderly patient population, and may help to maximize the clinical benefit from treatment while maintaining QoL.

## Supplementary Material

1

2

3

## Figures and Tables

**Table 1:** Patient Characteristics

Characteristic	Tepotinib (N = 255)
Median age, y (range)	72 (41–94)
Sex, n (%)	
Male	123 (48.2)
Female	132 (51.8)
Race, n (%)	
White	171 (67.1)
Asian	72 (28.2)
Black or African American	3 (1.2)
Other	1 (0.4)
Missing	8 (3.1)
ECOG PS, n (%)	
0	71 (27.8)
1	184 (72.2)
Smoking history, n (%)	
Never smoker Current or former smoker Missing	124 (48.6) 121 (47.5) 10 (3.9)
Histologic subtype, n (%) Adenocarcinoma 207 Squamous Sarcomatoid Adenosquamous Other	(81.2) 25 (9.8) 6 (2.4) 6 (2.4) 11 (4.3)
Prior anticancer therapy, n (%) Treatment-naïve Previously treated	125 (49.0) 130 (51.0)
Identification of *MET* ex14 skipping, n (%) ^a^ Liquid biopsy Tissue biopsy	156 (61.2%) 155 (60.8%)

Abbreviations: ECOG PS Eastern Cooperative Oncology Group performance status; *MET*ex14 *MET* exon 14.

a Patients could have *MET*ex14 skipping detected by both methods.

## Clinical Practice Points

- Tepotinib demonstrated durable clinical activity and a manageable safety profile in patients with *MET*ex14 skipping NSCLC in the phase II VISION trial
- To support physicians using tepotinib in the clinic, we performed detailed analyses of AEs of clinical interest in VISION, including time to first onset and resolution, and management through treatment modification
- Overall, tepotinib was well tolerated, with mostly mild/moderate AEs that were manageable without treatment discontinuation in this predominantly elderly population
- Edema, the most common AE of clinical interest and a MET inhibitor class effect, occurred in 69.8% of patients, was mostly mild/moderate (grade 3, 9.4%; grade ≥4, 0%), and was usually manageable with dose reduction (18.8%), treatment interruption (23.1%), and/or supportive measures
- Edema tended to occur early, may show a gradual onset, and was slow to resolve
- Proactive monitoring is critical to allow early recognition of edema and intervention, before it becomes more established, and challenging to manage
- In addition to conservative measures (eg support stockings, limb elevation, physical activity, and kinesiotherapy), dose reduction and/or treatment interruption (including frequent, short treatment breaks) appear to be the best current options for managing edema and should be implemented early
- As mild/moderate creatinine increases were common with tepotinib, with no evidence for an association with renal impairment, creatinine-independent measures of glomerular filtration rate may help avoid unnecessary dose modifications
- Mild/moderate nausea, diarrhea, and vomiting were common early during treatment, but tended to resolve in the first weeks, and may be mitigated by taking tepotinib with food and standard antidiarrheals/antiemetics

## Abbreviations:

ALT: alanine aminotransferase
AST: aspartate aminotransferase
BMI: body mass index
ECOG PS: Eastern Cooperative Oncology Group performance status
GI: gastrointestinal
HGF: hepatocyte growth factor
ILD: interstitial lung disease
MedDRA: Medical Dictionary for Regulatory Activities
*METex*14: *MET* exon 14
NSCLC: non-small cell lung cancer
QD: once daily
QoL: quality of life
SAE: serious adverse event
TKI: tyrosine kinase inhibitor
VEGF: vascular endothelial growth factor
