# Supplementary material for: Safety of Tepotinib in Patients With MET Exon 14 Skipping NSCLC and Recommendations for Management
Source: Clin Lung Cancer. Author manuscript; Available in PMC 2023 Apr 3. (PMC10068910; doi:10.1016/j.cllc.2022.03.002)
Supplement: 1 [file NIHMS1881578-supplement-1.pptx]

## Slide 1
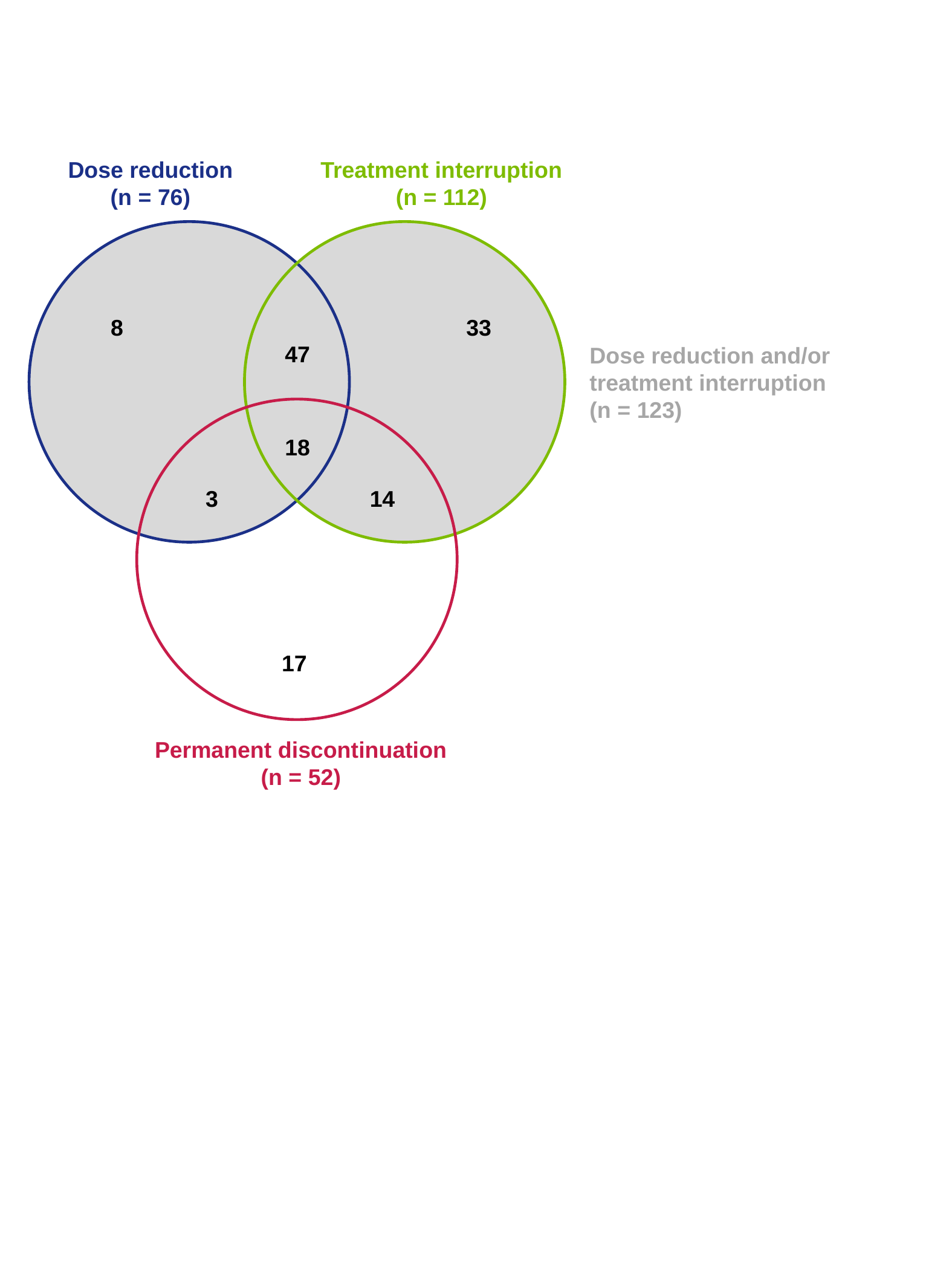

Dose reduction
(n = 76)
Treatment interruption
(n = 112)
8
33
47
Dose reduction and/or treatment interruption (n = 123)
18
3
14
17
Permanent discontinuation
(n = 52)
