# Supplementary figures and images for: Safety of Tepotinib in Patients With MET Exon 14 Skipping NSCLC and Recommendations for Management
Source: Clin Lung Cancer. Author manuscript; Available in PMC 2023 Apr 3. (PMC10068910; doi:10.1016/j.cllc.2022.03.002)

## Slide 1
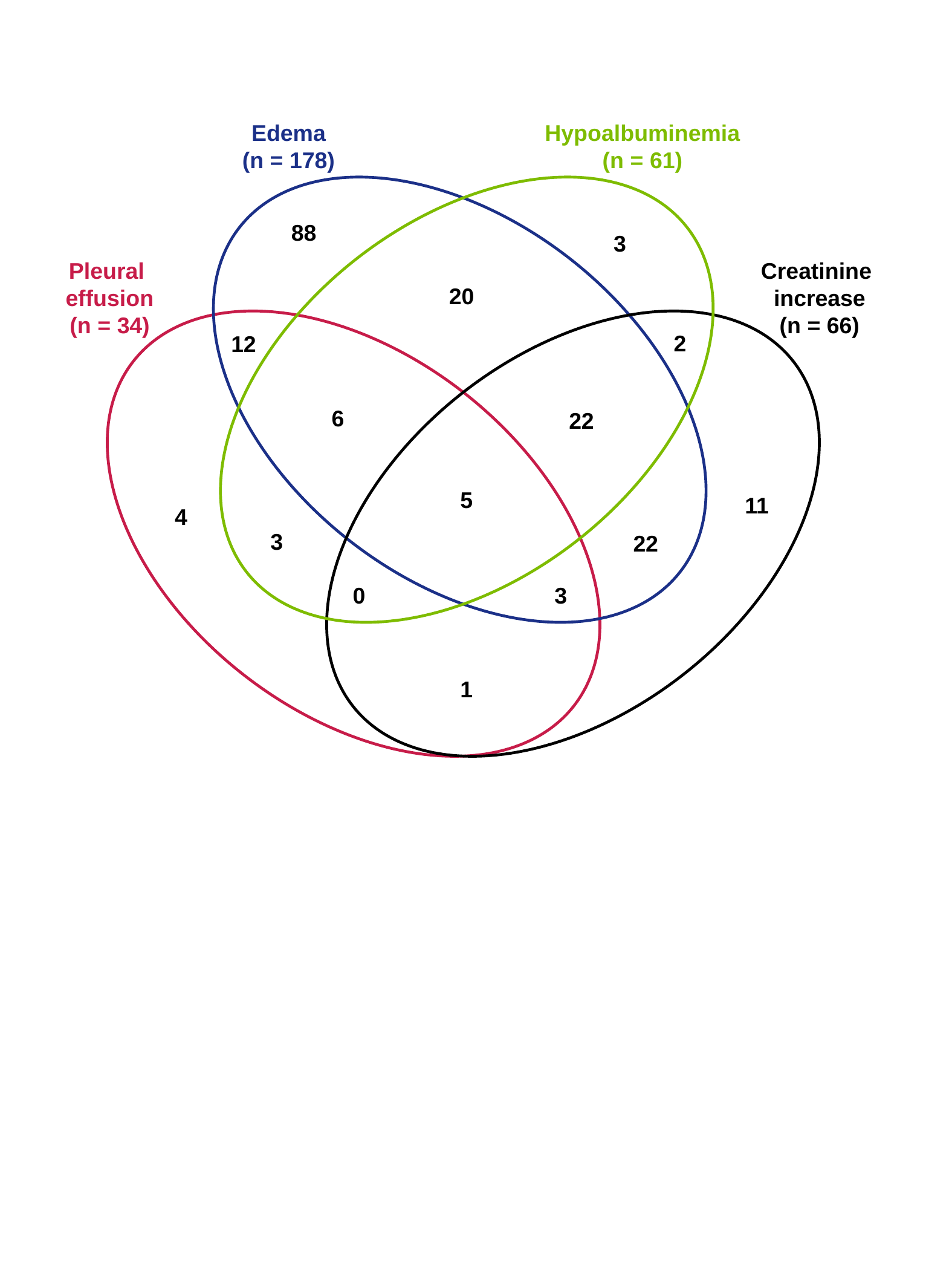

Edema
(n = 178)
Hypoalbuminemia
(n = 61)
88
3
Pleural
effusion
(n = 34)
Creatinine
increase
(n = 66)
20
2
12
6
22
5
11
4
3
22
3
0
1

Supplement: 2 [file NIHMS1881578-supplement-2.pptx]
