# Supplementary material for: Safety of Tepotinib in Patients With MET Exon 14 Skipping NSCLC and Recommendations for Management
Source: Clin Lung Cancer. Author manuscript; Available in PMC 2023 Apr 3. (PMC10068910; doi:10.1016/j.cllc.2022.03.002)
Supplement: 3 [file NIHMS1881578-supplement-3.docx]

**Supplementary Table 1.** Preferred terms included in the definitions of composite events.

| **Composite event** | **MedDRA preferred terms** |
| --- | --- |
| Edema | Face edema, edema, edema peripheral, localized edema, edema genital, periorbital edema, scrotal edema, peripheral swelling, abdominal wall edema, generalized edema |
| Hypoalbuminemia | Blood albumin abnormal, blood albumin decreased, hypoalbuminemia |
| Creatinine increase | Hypercreatininemia, blood creatinine increased, blood creatinine abnormal |
| ALT and/or AST increase | ALT abnormal, ALT increased, AST abnormal, AST increased, transaminases increased, transaminases abnormal, hypertransaminasemia, AST/ALT ratio abnormal |

Abbreviations: ALT = alanine aminotransferase; AST = aspartate aminotransferase; MedDRA = Medical Dictionary for Regulatory Activities.

# Supplementary Table 2. All-cause AEs leading to permanent discontinuation.^a^

| **Patients, n (%)** | **Tepotinib  (N = 255)** |
| --- | --- |
| Peripheral edema | 9 (3.5) |
| Pleural effusion | 5 (2.0) |
| Disease progression | 4 (1.6) |
| Dyspnea | 4 (1.6) |
| General physical health deterioration | 4 (1.6) |
| Genital edema | 3 (1.2) |
| Pneumonitis | 3 (1.2) |
| Blood creatinine increased | 2 (0.8) |
| Pneumonia | 2 (0.8) |
| Spinal fracture | 2 (0.8) |

^a^The following all-cause AEs each led to treatment discontinuation in 1 patient (0.4%): abdominal pain; acute myocardial infarction; acute respiratory failure; cardiac failure; cardiac tamponade; cardio-respiratory arrest; death; diarrhea; dysphagia; edema; electrolyte imbalance; embolism; face edema; headache; interstitial lung disease; localized edema; lung disorder; mental status changes; mucosal inflammation; nausea; neoplasm progression; pneumothorax; pulmonary embolism; pulmonary hemorrhage; respiratory tract infection; scrotal edema; spinal cord compression; and subdural hematoma.

Abbreviations: AE = adverse event.

# Supplementary Table 3. Incidence of all-cause edema, hypoalbuminemia, creatinine increase, and ALT/AST increase (composite terms), and constituent preferred terms (N = 255).

| **Patients, n (%)** | **Tepotinib  (N = 255)** | | |
| --- | --- | --- | --- |
|  | **All grades** | **Grade 3** | **Grade 4** |
| Edema (composite term) | 178 (69.8) | 24 (9.4) | 0 |
| Peripheral edema | 153 (60.0) | 20 (7.8) | 0 |
| Edema | 18 (7.1) | 0 | 0 |
| Generalized edema | 13 (5.1) | 5 (2.0) | 0 |
| Face edema | 7 (2.7) | 0 | 0 |
| Localized edema | 6 (2.4) | 1 (0.4) | 0 |
| Genital edema | 6 (2.4) | 3 (1.2) | 0 |
| Periorbital edema | 2 (0.8) | 0 | 0 |
| Scrotal edema | 2 (0.8) | 0 | 0 |
| Peripheral swelling | 1 (0.4) | 0 | 0 |
| Hypoalbuminemia (composite term) | 61 (23.9) | 14 (5.5) | 0 |
| Blood albumin decreased | 4 (1.6) | 0 | 0 |
| Hypoalbuminemia | 59 (23.1) | 14 (5.5) | 0 |
| Creatinine increase (composite term) | 66 (25.9) | 1 (0.4) | 0 |
| Blood creatinine increased | 64 (25.1) | 1 (0.4) | 0 |
| Hypercreatininemia | 2 (0.8) | 0 | 0 |
| ALT and/or AST increase (composite term) | 31 (12.2) | 6 (2.4) | 2 (0.8) |
| ALT increased | 29 (11.4) | 6 (2.4) | 2 (0.8) |
| AST increased | 19 (7.5) | 2 (0.8) | 1 (0.4) |
| Hypertransaminasemia | 1 ( 0.4) | 0 | 0 |

Abbreviations: ALT = alanine aminotransferase; AST = aspartate aminotransferase.

**Supplementary Figure 1.** Venn diagram showing the numbers of patients with all-cause AEs leading to tepotinib dose reduction, treatment interruption, and/or permanent discontinuation (n = 140). The black numbers within the segments refer to the number of patients with each type of dose modification or combination of dose modifications. Abbreviations: AE = adverse event.

**Supplementary Figure 2.** Venn diagram showing extent of overlap between patients with all-cause edema, hypoalbuminemia, pleural effusion, and/or creatinine increase events, irrespective of the event timing. The black numbers within the segments refer to the number of patients with each event or combination of events.
